# Genetic analyses of live weight and carcass composition traits in purebred Texel, Suffolk and Charollais lambs

**DOI:** 10.1017/S1751731119002908

**Published:** 2020-05

**Authors:** S. Fitzmaurice, J. Conington, N. Fetherstone, T. Pabiou, K. McDermott, E. Wall, G. Banos, N. McHugh

**Affiliations:** 1 Department of Animal and Veterinary Sciences, Scotland’s Rural College (SRUC), Easter Bush, Midlothian, Scotland EH25 9RG, UK; 2 Department of Animal and Biosciences, Teagasc, Animal & Grassland Research and Innovation Centre, Moorepark, Fermoy, P61 P203 Co. Cork, Ireland; 3 Sheep Ireland, Highfield House, Shinagh, Bandon, P72 X050 Co. Cork, Ireland

**Keywords:** sheep, Ireland, parameters, growth, muscle

## Abstract

Lamb live weight is one of the key drivers of profitability on sheep farms. Previous studies in Ireland have estimated genetic parameters for live weight and carcass composition traits using a multi-breed population rather than on an individual breed basis. The objective of the present study was to undertake genetic analyses of three lamb live weight and two carcass composition traits pertaining to purebred Texel, Suffolk and Charollais lambs born in the Republic of Ireland between 2010 and 2017, inclusive. Traits (with lamb age range in parenthesis) considered in the analyses were: pre-weaning weight (20 to 65 days), weaning weight (66 to 120 days), post-weaning weight (121 to 180 days), muscle depth (121 to 180 days) and fat depth (121 to 180 days). After data edits, 137 402 records from 50 372 lambs across 416 flocks were analysed. Variance components were derived using animal linear mixed models separately for each breed. Fixed effects included for all traits were contemporary group, age at first lambing of the dam, parity of the dam, a gender by age of the lamb interaction and a birth type by rearing type of the lamb interaction. Random effects investigated in the pre-weaning and weaning weight analyses included animal direct additive genetic, dam maternal genetic, litter common environment, dam permanent environment and residual variances. The model of analysis for post-weaning, muscle and fat depth included an animal direct additive genetic and litter common environment effect only. Significant direct additive genetic variation existed in all cases. Direct heritability for pre-weaning weight ranged from 0.14 to 0.30 across the three breeds. Weaning weight had a direct heritability ranging from 0.17 to 0.27 and post-weaning weight had a direct heritability ranging from 0.15 to 0.27. Muscle and fat depth heritability estimates ranged from 0.21 to 0.31 and 0.15 to 0.20, respectively. Positive direct correlations were evident for all traits. Results revealed ample genetic variation among animals for the studied traits and significant differences between breeds to suggest that genetic evaluations could be conducted on a per-breed basis.

## Implications

This study demonstrated the existence of genetic variation between different breeds of sheep for the three main live weight and two carcass composition traits in the Irish sheep production system suggesting that genetic evaluations should be conducted on a per-breed basis. This would allow for more informed and accurate selection decisions on farm, resulting in superior productivity and profitability within Irish sheep flocks.

## Introduction

Lamb live weight and the rate at which the animal grows have been defined as the key drivers of profitability in Irish (Byrne *et al.*, 2010) and international (Cocks *et al.*, 2002; Conington *et al.*, 2004; Jones *et al.*, 2004a) sheep production systems. In Ireland, for example, each additional day a lamb requires to reach its target slaughter weight results in an economic loss of €1.41 per lamb per day (Byrne *et al.*, 2010). In addition to the live weight traits, carcass composition also has an impact on the profitability of sheep production systems with an increase of one point, on the EUROP scale for muscle depth leading to an economic gain of €0.35 per lamb and an increase of one point on the fat scale leading to an economic loss of −€0.52 per lamb (Byrne *et al.*, 2010). Lamb live weight, weight gain and carcass composition have been shown to vary greatly not only across the various stages of a lambs’ growth period, such as pre- and post-weaning (Leymaster and Jenkins, 1993; Djemali *et al.*, 1994; Leeds *et al.*, 2012) but also across a plethora of breeds, including meat (Osorio-Avalos *et al.*, 2012), wool (Safari *et al.*, 2007) and dual purpose (Dixit *et al.*, 2001) breeds.

Previous research has shown considerable variability across both pre- and post-weaning lamb growth rates not only at a phenotypic level (Dixit *et al.*, 2001) but also at a genetic level (Safari *et al.*, 2005; Thiruvenkadan *et al.*, 2011), with heritabilities for lamb live weight at different ages ranging from 0.15 to 0.41 (Safari *et al.*, 2005). Such studies, however, have tended to focus on small sample sizes, which may not accurately represent the whole sheep population. Furthermore, although some studies have shown that genetic variability exists among breeds (Freking and Leymaster, 2004; Osorio-Avalos *et al.*, 2012), genetic parameters and sheep genetic evaluations in Ireland to date have been developed within a multi-breed population context (Pabiou *et al.*, 2014), and heretofore the genetic variation within individual breeds has not been considered.

The objective of the present study, therefore, was to estimate genetic parameters and breeding values for a range of lamb live weight and carcass composition traits within three breeds commonly recorded in Ireland namely Texel, Suffolk and Charollais. Results from the present study would determine differences between breeds in the genetic evaluations of sheep in Ireland.

## Material and methods

### Data

A full database was extracted across three breeds, namely Texel, Suffolk and Charollais, from Sheep Ireland, the Irish national database (http://www.sheep.ie). Records pertaining to years 2010 to 2017, inclusive, were retained for analyses. Only purebred lambs (as defined by the data records) of the three aforementioned breeds (i.e., Texel, Suffolk and Charollais) were considered in the present study.

In Ireland, lamb live weights are recorded at three time points post-lambing by Irish producers using weigh scales: pre-weaning, at weaning and post-weaning, the latter coinciding with muscle and fat ultrasound scanning. Based on the editing criteria used for the national genetic evaluations, pre-weaning weight was defined as live weight taken between 20 and 65 days of age; only records of lambs weighing between 12.00 and 32.00 kg were retained in the present study. Weaning weight was defined as the live weight recorded between 66 and 120 days of age and weighing between 20.00 and 55.00 kg. Post-weaning weight was defined as live weight measured between 121 and 180 days of age; only lambs with live weight records between 25.00 and 75.00 kg were considered for further analysis. Across all live weight measurements, average daily gain was calculated for each lamb with a known birth and weigh date at either of the three weight points; only average daily gains between 100 and 650 g/d were retained for each live weight measurement (261 lambs with an erroneous average daily gain were omitted from subsequent analyses). Muscle and fat depth traits were recorded on the same day as post-weaning weight in all lambs. Only muscle depth measurements within the range of 10 to 44 mm and fat depth measurements ranging within 1 to 23 mm were retained.

Live weight and carcass composition measurement records were discarded if flock of birth, sire, dam or maternal grandsire were unknown. Dams with no known parity number or a parity number >10 were discarded; parity number was subsequently categorised as 1, 2, 3, 4 or ≥ 5. Age at first lambing was defined based on the age of the ewe at first lambing; ewes were either defined as lambing for the first time as ewe lambs (between 8 and 18 months of age) or those that lambed for the first time as hoggets (between ≥18 and 28 months of age). Birth type was defined as the number of lambs born per lambing event; only birth types between 1 (singles) and 4 (quadruplets) were retained. Rearing type was defined as the number of lambs reared per litter; only rearing type between 1 and 3 were retained for analysis. Lambs that were recorded as artificially reared or reared by a non-genetic dam were not included for further analysis.

For all traits, each lamb was allocated to a contemporary group of breed-by-flock-by-week of weighing. Only contemporary groups containing at least five records were retained for analysis. Following all edits described above, 33 721 pre-weaning weight records, 32 623 weaning weight records, 28 140 post-weaning weight records, 21 468 muscle depth records and 21 442 fat depth records were retained for genetic analysis; the breakdown of records per breed is shown in Table 1.

**Table 1:** Number of lambs (*n*), trait mean (*µ*), SD, CV, corresponding mean lamb age, and number of sires, dams, maternal grandsires (MGS), flocks and contemporary groups (CGs) by trait and breed

Trait (units of measurement)	Breed	*n*	*µ* (SD)	Age	CV (%)	Sires	Dams	MGS	Flocks	CGs
Pre-weaning weight (kg)	Texel	11 891	20.86 (4.70)	46.59	22.53	804	5359	1093	162	480
	Suffolk	8783	22.32 (4.85)	45.12	21.73	541	3816	759	110	329
	Charollais	13 047	20.58 (4.58)	46.20	22.25	602	4965	919	139	456
Weaning weight (kg)	Texel	12 388	36.69 (7.63)	96.92	20.80	847	5688	1176	161	508
	Suffolk	7839	40.93 (7.87)	96.31	19.23	542	3625	774	107	308
	Charollais	12 396	37.09 (7.40)	96.65	19.95	607	4820	913	139	449
Post-weaning weight (kg)	Texel	12 074	48.70 (9.47)	144.76	19.45	847	5746	1179	161	422
	Suffolk	6819	56.42 (10.79)	147.24	19.12	508	3411	753	96	281
	Charollais	9247	51.92 (9.91)	148.99	19.09	567	4106	844	129	354
Muscle depth (mm)	Texel	8810	32.59 (4.09)	146.57	12.55	662	4259	916	108	280
	Suffolk	5589	34.11 (5.01)	151.28	14.69	402	2792	621	69	204
	Charollais	7094	33.23 (3.97)	151.81	11.95	455	3344	714	96	252
Fat depth (mm)	Texel	8782	6.10 (2.70)	146.63	44.26	661	4250	916	108	281
	Suffolk	5556	8.50 (4.00)	151.42	47.06	399	2784	618	69	205
	Charollais	7087	8.10 (3.80)	151.82	46.91	455	3346	712	97	253

### Genetic analysis

Variance components were estimated for each lamb live weight trait (i.e., pre-weaning, weaning and post-weaning weights) and each carcass composition trait (i.e., muscle depth and fat depth) using linear mixed animal models in ASReml (Gilmour *et al.*, 2009) separately for each breed. The model employed was:
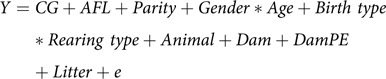
where *Y* = lamb live weight or carcass composition record, CG = contemporary group, AFL = age at first lambing of the dam, Parity = parity of the dam, Gender*Age = the interaction between the gender and age of the lamb, Birth type*Rearing type = the interaction between the birth type and rearing type of the lamb, Animal = random animal direct additive genetic effect, Dam = random maternal genetic effect, DamPE = random dam permanent environmental effect associated with multiple lambing records of the same dam, Litter = common environmental effect reflecting the non-genetic covariance among members of the same litter and e = random residual effect.

Each model was progressively built up from including just a residual effect to include a direct genetic, maternal genetic, dam permanent environmental and litter common environmental effect. In the case of post-weaning weight, muscle and fat depth, the model included a direct genetic and a litter common environmental effect only as there was no significant dam effect. A log-likelihood ratio test was used to determine if the additional random terms improved the fit of the data (Ferreira *et al.*, 1999). While the maternal genetic and dam permanent environmental effect were not always significant, these effects were kept in the model as the log-likelihood ratio test suggested it was the model of best fit.

Direct heritability was calculated as the ratio of the direct additive genetic variance to the observed total phenotypic variance. Maternal heritability was estimated as the ratio of the maternal genetic variance to the total phenotypic variance. Common environmental effect was calculated as the ratio of the litter variance to the total phenotypic variance. Dam repeatability was calculated as the ratio of maternal genetic variance plus permanent environment to the total phenotypic variance. The correlation between the direct additive and maternal genetic effects was also estimated where applicable. Genetic correlations between the studied traits were estimated pairwise using the model previously described in a series of bivariate analyses. Estimated breeding values (**EBVs**) were calculated for each trait and genetic trends were produced from these results by estimating the slope of the average ram EBV per year of birth. Genetic trends were only produced for sires with at least 10 progeny and ranged from 3 to 61 sires per year across all traits and breeds.

## Results

### Phenotypic values and data structure

Edited data used in the genetic analyses are shown in Table 1. The Suffolk breed proved to be the heaviest at all three live weight measurements although they were slightly younger at both pre-weaning and weaning weights. The Suffolk breed also had the highest muscle and fat depth among the three breeds studied although this may be attributed partly to the higher weight at scanning. Overall, the Texel breed had the highest number of records across all five traits and they also had the highest number of flocks. Judging on the CV, the greatest variability was observed in fat depth and the least variability was observed for muscle depth, and this was true across all breeds.

### Genetic parameters

Variance components were estimated (Table 2 and heritability estimates were subsequently derived for each trait and breed. All estimates of genetic SD and direct heritability were statistically greater than zero (*P* < 0.05) as shown in Table 3. All traits studied apart from pre-weaning weight were most heritable in the Texel breed. Pre-weaning weight was most heritable in the Suffolk breed. Maternal heritability was significantly greater than zero for all weight traits in the Texel breed, pre-weaning weight in Suffolks and weaning weight in Charollais. The litter common environmental effect accounted for the majority of the total phenotypic variance for most live weight traits and a significant proportion for the carcass composition traits.

**Table 2:** Lamb direct genetic variance (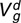), maternal genetic variance (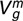), variance due to common environmental effect (C_m_) and variance due to maternal repeatability (PE_m_) per trait and breed; model of analyses of post-weaning weight, muscle and fat depth did not include a maternal effect

	Breed	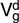 (SE)	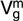 (SE)	C_m_ (SE)	PE_m_ (SE)
Pre-weaning weight	Texel	1.57 (0.27)*	0.58 (0.18)*	2.98 (0.19)*	0.57 (0.19)*
	Suffolk	2.44 (0.40)*	0.56 (0.22)*	3.39 (0.24)*	0.12 (0.23)
	Charollais	1.39 (0.25)*	0.20 (0.13)	3.54 (0.18)*	0.06 (0.16)
Wean weight	Texel	6.89 (0.81)*	0.98 (0.39)*	6.55 (0.48)*	0.43 (0.43)
	Suffolk	4.79 (1.03)*	0.84 (0.55)	7.85 (0.73)*	0.26 (0.64)
	Charollais	5.77 (0.79)*	0.87 (0.39)*	6.01 (0.45)*	0.18 (0.41)
Post-weaning weight	Texel	11.94 (1.10)*		8.99 (0.62)*	
	Suffolk	7.42 (1.48)*		11.55 (1.09)*	
	Charollais	6.79 (1.03)*		8.73 (0.74)*	
Muscle depth	Texel	2.76 (0.28)*		1.39 (0.18)*	
	Suffolk	2.05 (0.35)*		1.48 (0.26)*	
	Charollais	1.70 (0.25)*		1.51 (0.18)*	
Fat depth	Texel	0.01 (0.00)*		0.01 (0.00)*	
	Suffolk	0.01 (0.00)*		0.02 (0.00)*	
	Charollais	0.01 (0.00)*		0.01 (0.00)*	

SE = standard error of estimate.

* Estimates significantly different (*P* < 0.05) from zero.

**Table 3:** Lamb direct heritability (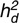), maternal heritability (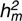), proportion of phenotypic variance due to the common environmental effect (C^2^m), maternal repeatability (R_m_), and the correlation between direct and maternal genetic effects (CORR d/m) per trait and breed; model of analyses of post-weaning weight, muscle and fat depth did not include a maternal effect

	Breed	h^2^d (SE)	h^2^m (SE)	C^2^m (SE)	Rm (SE)	CORR d/m (SE)
Pre-weaning weight	Texel	0.16 (0.03)*	0.06 (0.02)*	0.30 (0.02)*	0.12 (0.02)*	−0.65 (0.07)*
	Suffolk	0.22 (0.03)*	0.05 (0.02)*	0.31 (0.02)*	0.06 (0.02)*	−0.77 (0.06)*
	Charollais	0.14 (0.02)*	0.02 (0.01)	0.35 (0.02)*	0.03 (0.01)	−0.84 (0.05)*
Wean weight	Texel	0.27 (0.03)*	0.04 (0.02)*	0.26 (0.02)*	0.06 (0.02)*	−0.61 (0.07)*
	Suffolk	0.17 (0.03)*	0.03 (0.02)	0.27 (0.02)*	0.04 (0.02)	−0.68 (0.09)*
	Charollais	0.23 (0.03)*	0.03 (0.02)*	0.24 (0.02)*	0.04 (0.01) *	−0.71 (0.06)*
Post-weaning weight	Texel	0.32 (0.03)*		0.24 (0.02)*		
	Suffolk	0.16 (0.03)*		0.25 (0.02)*		
	Charollais	0.18 (0.03)*		0.23 (0.02)*		
Muscle depth	Texel	0.31 (0.03)*		0.16 (0.02)*		
	Suffolk	0.21 (0.03)*		0.15 (0.03)*		
	Charollais	0.21 (0.03)*		0.19 (0.02)*		
Fat depth	Texel	0.20 (0.03)*		0.20 (0.02)*		
	Suffolk	0.15 (0.03)*		0.17 (0.03)*		
	Charollais	0.17 (0.03)*		0.17 (0.02)*		

SE = standard error of estimate.

* Estimates significantly different (*P* < 0.05) from zero.

Negative correlations were estimated between direct additive and maternal genetic effects within trait for all breeds (Table 3). This is an antagonistic correlation suggesting that animals with genetically superior direct additive genetic effect are expected to be maternally inferior. Significant (*P* < 0.05) positive genetic correlations between the direct additive genetic effects on pre-weaning and subsequent weights for each of the three breeds were calculated (Table 4). Direct genetic correlations between live weight traits and the two carcass composition traits were also strongly positive reaching a maximum of 0.72 (±0.04) between weaning weight and muscle depth for the Texel breed (Table 4).

**Table 4:** Lamb genetic correlations (standard error in parentheses) between the direct additive genetic effects for each trait (below the diagonal) and the maternal genetic effects for each trait (above the diagonal) by breed; model of analyses of post-weaning weight, muscle and fat depth did not include a maternal effect

	Trait	Pre-weaning	Weaning	Post-weaning	Muscle depth
Texel	Pre-weaning		0.95 (0.03)*		
	Weaning	0.76 (0.04)*			
	Post-weaning	0.65 (0.07)*	0.94 (0.02)*		
	Muscle depth	0.57 (0.06)*	0.72 (0.04)*	0.69 (0.03)*	
	Fat depth	0.31 (0.08)*	0.49 (0.07)*	0.45 (0.06)*	0.42 (0.06)*
Suffolk	Pre-weaning		0.80 (0.06)*		
	Weaning	0.61 (0.09)*			
	Post-weaning	0.76 (0.08)*	0.77 (0.07)*		
	Muscle depth	0.41 (0.09)*	0.23 (0.15)	0.61 (0.07)*	
	Fat depth	0.36 (0.11)*	0.27 (0.16)	0.29 (0.12)*	0.48 (0.09)*
Charollais	Pre-weaning		0.97 (0.04)*		
	Weaning	0.55 (0.07)*			
	Post-weaning	0.63 (0.07)*	0.90 (0.04)*		
	Muscle depth	0.51 (0.08)*	0.63 (0.07)*	0.54 (0.06)*	
	Fat depth	0.18 (0.10)	0.27 (0.10)*	0.26 (0.09)*	0.41 (0.08)*

* Estimates significantly different (*P* < 0.05) from zero.

### Genetic trends

**Figure 1:**
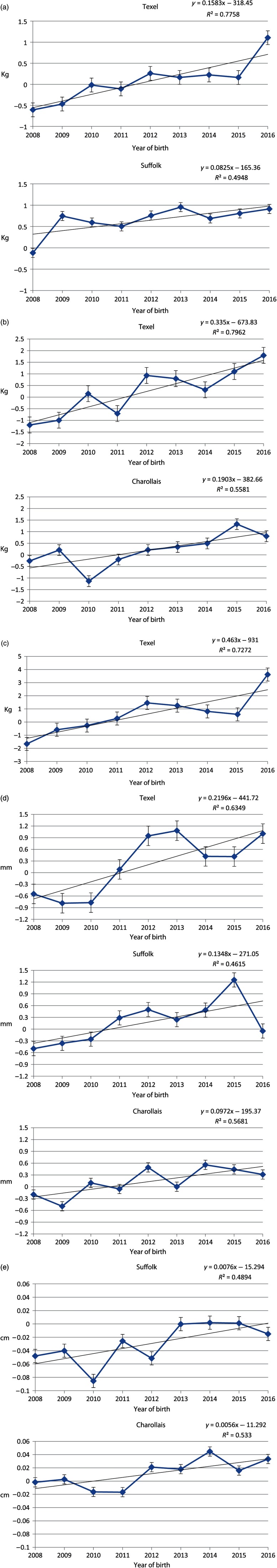
Significantly different from zero (*P* < 0.05) genetic trends of estimated breeding values of rams (standard errors shown in error bars) for (a) pre-weaning weight, (b) weaning weight (c) post-weaning weight (d) muscle depth and (e) fat depth.

Genetic trends based on EBVs of rams with ≥10 progeny (Figure 1) indicate that positive genetic gain is occurring in all live weight traits. Significant (*P* < 0.05) trends were observed for all live weight traits in the Texel breed, pre-weaning weight in the Suffolk breed and weaning weight in the Charollais breed. Muscle depth had a strong positive significant trend for all breeds, while fat depth had weakly positive significant trends for both the Suffolk and Charollais breeds. There was considerable variation in genetic trends estimated for the same trait among the three studied breeds with higher rates of genetic gain being achieved in the Texel breed for live weight traits and muscle depth in comparison to the other two breeds.

## Discussion

Live weight measurements on lambs are among the key performance indicators in profitable sheep production systems. To date, most genetic studies undertaken in Ireland have tended to estimate genetic parameters for lamb live weight and carcass composition traits simultaneously across a range of breeds rather than investigating on an individual breed basis. Therefore, in the present study, we investigated if estimates of genetic parameters and breeding values differed between breeds within the Irish sheep population when the breeds were evaluated on a within-breed basis. Results showed significant differences in additive genetic variance and direct heritability of each trait between the Texel, Suffolk and Charollais breeds, warranting within-breed genetic analyses.

### Phenotypic values

In comparison to previous studies conducted on an Irish sheep population, lamb live weight in the present study was greater for all three live weight traits examined. Previously pre-weaning, weaning and post-weaning weights in Irish purebred lambs were shown to be 19.64, 33.00 and 48.00 kg, respectively (McHugh *et al.*, 2016; McHugh *et al.*, 201*7*). The increased live weight observed in the current study may be attributed to the fact that only terminal purebred lambs were examined, whereas maternal and cross-bred lambs had been also included in the previous studies. The carcass composition traits in the present study showed similar results to those previously reported in the literature for purebred Irish lambs. An earlier study conducted in Ireland (O’Brien *et al.*, 2016) showed a mean of 33.21and 7.55 mm for muscle and fat depth traits, respectively. The first study carried out in the UK on live weight and carcass composition traits in terminal sire sheep was reported by Simm and Dingwall (1989) from which selection indices for terminal sire breeds were implemented in practice for the UK sheep industry and responses to selection reported. Jones *et al.* (2004b) reported similar findings to the present study for post-weaning weight, muscle depth and fat depth traits for the three breeds studied in terms of breed ranking; however, fat depth proved to be considerably higher in the present study. Other studies have been reported for cross-bred and hill lambs (Merrell *et al.*, 1990; Conington *et al.*, 2004). Again these findings were very similar to the present study for the post-weaning weight and muscle depth values; however, fat depth proved to be higher for all breeds in the present study although the ranking of the breeds remained the same. Merrell *et al.* (1990) reported weight at slaughter for Suffolk, Texel and Charollais cross-bred lambs in the UK, which was recorded at a similar age to post-weaning weight in the present study, ranging from 39.50 kg (Texel) to 41.10 kg (Suffolk). Although these lambs were lighter than those in the present study, the ranking of breeds was similar with the Suffolk breed having the highest live weight and the Texel breed having the lowest post-weaning live weight. Throughout the rest of the world, many studies have recorded live weight in lambs at different time points; however, few of these studies have focused on the breeds investigated in the current study (Safari and Fogarty, 2003), although Shrestha *et al.* (1985) reported similar findings for pre-weaning and weaning weights in Canadian Suffolks. Furthermore, a US study of Texel- and Suffolk-sired cross-bred lambs (Leymaster and Jenkins, 1993) showed similar live weight results to the present study with the Suffolk breed proving to be the heaviest at both weaning and post-weaning weights in comparison to the Texel breed. One contrast observed in Leymaster and Jenkins’ (1993) study compared to the present study was that the Suffolk and Texel breeds were recorded to have the same mean weight for pre-weaning weight, whereas in the present study the Suffolk is considerably heavier for all live weights; however, this may be attributed to the multiple-rearing environment having a greater effect on the growth potential of the Suffolk lambs over the Texel lambs.

Many of the studies on carcass composition previously conducted are not comparable to the present study due to different methods used and time points of measurement (Safari and Fogarty, 2003). Many of these studies tended to measure both muscle and fat depth at a later time point with the majority measured when the lamb is between 7 and 16 months of age (Safari and Fogarty, 2003). However, one study conducted by Jones *et al.* (2004b) showed very similar results to the present study with the Suffolk breed having the highest muscle and fat depth and the Texel breed having the lowest fat depth out of the three studied breeds.

### Genetic parameters

Direct and maternal heritability estimates reported in the present study for live weight and carcass composition traits are all within the ranges previously reported in the literature. Within the present study with the exception of pre-weaning weight and fat depth, direct heritability differed substantially among breeds for all traits analysed with most variability observed in the post-weaning weight trait where direct heritability ranged from 0.16 (Suffolk) to 0.32 (Texel). Genetic parameter estimates have not previously been reported in Ireland on a per-breed basis. One previous study reported genetic parameter estimates within a multi-breed analysis (McHugh *et al.*, 2017) including a heritability estimate for pre-weaning weight in Irish lambs of 0.09, which is lower than all pre-weaning weight estimates in the present study. This may be attributed to the differences between the breeds lowering the heritability in the previous study in comparison to the present study, which was conducted on genetically more homogeneous purebred populations. Higher accuracy of EBVs would also be expected in within-breed genetic evaluations as a result of increased direct heritability estimates. Maternal heritability estimates were low for all three live weight traits measured and were not significant for the two carcass composition traits. These results contrast significantly with the study on pre-weaning weight by McHugh *et al*. (2017) where a maternal heritability of 0.25 was reported in a multi-breed Irish sheep population. This difference may, however, be due to different models used in the analysis as much of the variation in the present study was due to the common environmental effect, which was not included in the study of McHugh *et al.* (2017). In the UK, previous studies have estimated genetic parameters for the Suffolk breed for all traits analysed in the present study (Maniatis and Pollott, 2002a and 2002b; Simm *et al.*, 2002) and results were generally similar. Simm *et al.* (2002) suggested that direct heritability estimates would increase with lamb age due to the lessening maternal influence and increased direct influence. This was indeed the case in the present study for Texel and Charollais breeds. For the Suffolk breed, however, the opposite was true as direct heritability decreased from 0.22 (pre-weaning) to 0.16 (post-weaning) while maternal heritability also decreased.

The strong positive direct genetic correlations among the three live weight traits were as expected, indicating that lambs that are genetically heavier early in life are also more likely to be genetically heavier later on. While these figures corresponded well with the literature, some of the estimates in the present study were outside the ranges previously reported with weaker correlations observed in the present study compared to those previously reported (Safari and Fogarty, 2003). This, however, may be due to the fact that few studies estimated genetic correlations between live weight traits at the specific times that were reported in the present study and may also be due to many of the previous studies being based in Australia or Asia where the studied breeds being differ greatly to those in the current study (Safari and Fogarty, 2003). Many of these studies also tended to have a far greater age spread between weight ages than those reported in the present study. No previous studies have investigated at genetic correlations among growth traits for the Texel or Charollais breeds, individually. However, there was one UK study by Simm *et al.* (2002) that showed the direct and maternal genetic correlations between pre-weaning and post-weaning weight for the Suffolk breed to be 0.69 and 0.86, respectively. These results were broadly in the range of those reported in the present study, although stronger maternal genetic correlations between the traits were recorded in the present study. The difference between the previous study and the present study may be attributed to the fact that the previous study (Simm *et al.*, 2002) was based on one flock only, whereas the present study includes the entire recorded population.

As with the live weight traits, strong positive correlations were also seen among the two carcass composition traits and post-weaning weight. Very few previous studies have estimated correlations among these traits at the similar time points to the present study; however, the direct correlations estimated here are broadly within the range previously reported (Atkins *et al.*, 1991; Simm *et al.*, 2002; Ingham *et al.*, 2003). These strong positive correlations indicate that by breeding for heavier lambs, we are also breeding for more muscular but also fatter lambs. The former is desirable but the latter undesirable. Although these traits are antagonistic, we need to aim to select for animals that are more muscular and less fat while still achieving live weight targets in order to maximise genetic gain and profitability. Appropriate selection indices need to be developed for this matter, optimally combining live weight and carcass traits.

For pre-weaning and weaning weight, a negative correlation was observed between the direct additive and maternal genetic effects. Although this corresponded with the majority of the literature for growth and live weight traits (Notter, 1998; Safari and Fogarty, 2003; Maxa *et al.*, 2007), previous studies have reported very mixed results with some positive correlations appearing also between live weight traits (Tosh and Kemp, 1994; Nasholm and Danell, 1996; Snyman *et al.*, 1996; Yazdi *et al.*, 1997; Rao and Notter, 2000). This variation of results previously reported in the literature may be indicative of differences in data structure but may also be due to breed differences (Maniatis and Pollott, 2002a). The antagonistic correlation reported between direct and maternal effects in the present study suggests that by selecting rams to breed heavier lambs, their daughters will have lighter lambs. In order to counteract this, optimal combination of antagonistic traits in a properly developed selection index is needed to support selection decisions.

### Genetic trends

To our knowledge, this is the first time genetic trends on Irish sheep are reported for the studied traits. Genetic trends varied between the three breeds for all traits in the present study. From the genetic trends, the Texel breed appears to be achieving the most genetic gain as significantly positive trends were recorded for all live weight traits as well as the muscle depth trait. No significant trend was found for fat depth in the Texels, indicating that this trait is remaining relatively static which is more desirable than the increasing trend observed for the Suffolk and Charollais breeds. The muscle depth trait showed a positive trend for all three breeds. These results are indicative of the ongoing genetic selection programme in Ireland based on the emphasis that is being placed on muscle depth for all breeds as well as the increase in genetic gain in live weight that has been seen in all three breeds.

## Conclusion

Variance components and genetic parameters derived in the present study for five live weight and carcass traits may be used to support the breeding programme of sheep in Ireland. Considerable differences in genetic analysis results were found between the Texel, Suffolk and Charollais breeds for each of the five traits examined in the present study. Differences were observed in both heritability and genetic correlation estimates suggesting that current genetic improvement systems may benefit by considering these breeds separately in future genetic evaluations.

## Software and data repository resources

None of the data were deposited in an official repository.
